# Automatic Sequential Stitching of High-Resolution Panorama for Android Devices Using Precapture Feature Detection and the Orientation Sensor

**DOI:** 10.3390/s23020879

**Published:** 2023-01-12

**Authors:** Yaseen, Oh-Jin Kwon, Jinhee Lee, Faiz Ullah, Sonain Jamil, Jae Soo Kim

**Affiliations:** 1 Department of Electronics Engineering, Sejong University, Seoul 05006, Republic of Korea; 2 College of Semiconductor System, Yonsei University, Wonju-si 26493, Republic of Korea

**Keywords:** mobile panorama, computer vision, sequential image stitching, smartphone’s gyroscope sensors, automatic panorama generation

## Abstract

Image processing on smartphones, which are resource-limited devices, is challenging. Panorama generation on modern mobile phones is a requirement of most mobile phone users. This paper presents an automatic sequential image stitching algorithm with high-resolution panorama generation and addresses the issue of stitching failure on smartphone devices. A robust method is used to automatically control the events involved in panorama generation from image capture to image stitching on Android operating systems. The image frames are taken in a firm spatial interval using the orientation sensor included in smartphone devices. The features-based stitching algorithm is used for panorama generation, with a novel modification to address the issue of stitching failure (inability to find local features causes this issue) when performing sequential stitching over mobile devices. We also address the issue of distortion in sequential stitching. Ultimately, in this study, we built an Android application that can construct a high-resolution panorama sequentially with automatic frame capture based on an orientation sensor and device rotation. We present a novel research methodology (called “Sense-Panorama”) for panorama construction along with a development guide for smartphone developers. Based on our experiments, performed by Samsung Galaxy SM-N960N, which carries system on chip (SoC) as Qualcomm Snapdragon 845 and a CPU of 4 × 2.8 GHz Kyro 385, our method can generate a high-resolution panorama. Compared to the existing methods, the results show improvement in visual quality for both subjective and objective evaluation.

## 1. Introduction

Smartphone devices have a limited field of view (FoV) of 77° [1]. To provide a more immersive experience of the scene, panoramic images are generated from a sequence of images with a sufficient area of overlap between consecutive image frames. The panoramic application allows users to capture 360° views of the surrounding area from a single viewpoint and seamlessly combine multiple images.

Due to the recent developments in mobile phone hardware, mobile camera resolution and computing power have greatly increased. Hence, users are now seeking instant panorama construction with high resolution [1].

A simple concatenation of images with overlapping areas to form panoramic image results in visible seams due to variations in the angle viewpoint of the camera and scene illumination, along with the spatial position errors of the images. Image stitching algorithms can be used to find optimal seems in overlapping areas between two consecutive images and generate a final panorama by merging these images along the seams with minimal margining artifacts [2]. Since the panorama generation process requires a substantial amount of computational power, and smartphone devices are computationally less powerful than desktop PCs for image processing, an efficient algorithm is needed to enable high-resolution panorama generation for mobile devices. The developer may also want to embed privacy and security data such as watermarks and signatures into their panoramic images. Based on user requirements and functionality, developers need to build customized panorama applications. Although some smartphones are equipped with built-in panorama applications, their implementation details are not accessible to programmers. To build a customized automatic panoramic application with high resolution, several challenges remain in the development process. Issues that can be faced by a developer in developing an automatic stitching program include (a) stitching failure; (b) event synchronization with the orientation sensor (to avoid deadlocks); (c) performance challenges (to avoid memory overflow) and the synchronization of several modules relative to each other; and (d) the distortion of interim panoramas. In sequential stitching, distortion is inevitable, and we address this issue in our Sense-Panorama method (see Section 3). For the reader’s convenience, we will refer to our proposed method as “Sense-Panorama”, as this method is able to fully automate the panorama-capturing process using the orientation sensor and precapture feature detection module.

Similarly, triggering and controlling events with the orientation sensor of the device is a challenging task. Stitching involves computationally complex algorithms such as feature matching and homography estimation. Hence, efficient techniques are presented in this study to address the challenges discussed. The issue of stitching failure and distortion in sequential stitching is solved using “Sense-Panorama”. In sequential stitching, two consecutive frames are taken by the camera, and the images are stitched together while exploiting control from the orientation sensor. The resulting interim panorama is immediately displayed to the user. This interim panorama is then extended with the next image frame to obtain the second interim panorama. This process continues until the capture session is completed. If the number of features in the overlapping area between the two consecutive image frames is not sufficient, stitching failure will occur. The goal of this paper is to automatically generate a high-resolution panorama on mobile devices by addressing the issues of stitching failure and distortion removal.

### 1.1. Contributions

List of contribution

1. Develop an Android-based application
2. Address the issue of stitching failure
3. Address the issue of distortion
4. Control events with an orientation sensor for automation

### 1.2. Organization

The remainder of this paper is organized as follows. In Section 2, we succinctly discuss feature-based image stitching. Section 3 describes the proposed method “Sense-Panorama” which includes the operation of the orientation sensor and the precapture feature detection technique, Section 4 presents the experimental results, and Section 5 concludes our work.

## 2. Background

Image stitching involves computationally complex algorithms such as feature detection, feature matching, outlier removal, and homography estimation, which causes performance issues when running such algorithms on mobile devices [1,2]. Several requirements and challenges must be kept in mind while building an automatic, sequential panorama generative application for mobile devices. In automatic panorama generation, the user slowly moves their device in front of the scene and covers a rotation angle covering a 0° to 360° range around a fixed standing position. In this process, the orientation sensor is used to detect motion and take pictures. These pictures are sequentially stitched together and displayed to the user instantly. Several issues can be faced by developers when developing a panorama application. Such issues include sequential stitching failure (unable to proceed with the stitching process when two consecutive frames could not be stitched due to less number of local feature points, we present a novel method called “Sense-Panorama” to address this issue, deadlock ( several modules of the application need to be responsive to each other on time as some modules are implemented in Java and some are in C++ when they failed to respond in time, the application goes to a deadlock state), a memory overflow (we also need to clear memory from the data that is not used in the remaining session of the program such as images frames and preview frames from the camera), and distortion in the panorama. Our Android-based application consists of several modules (C++ and Java based), if they are not categorized into synchronous and asynchronous tasks, issues of deadlock and memory overflow are inevitable. This issue is due to the fact that image processing tasks need to be executed using C++. The front-end modules are implemented in Java. Executing these modules without synchronizing, will lead to deadlocks and memory overflow as we have experienced during our experiments. The process would not proceed further and was abandoned by the Android OS.

Distortion is inevitable in sequential stitching when each previously created interim panorama is stitched together with the next frame. All these issues have been addressed. The performance issue is very critical when the device does not respond in real-time and causes failure, as several submodules, such as the user interface, camera device for live preview, gyroscope for device orientation, and panorama stitching, need to be executed synchronously. In this work, we address all the above-mentioned issues and present Sense-Panorama to solve stitching failure and remove distortion. In the first subsection, we briefly study how image stitching works, and in the second subsection, we discuss how data can be processed from a gyroscope to control our proposed image stitching application.

### 2.1. Image Stitching

Image stitching can be broadly classified into three main categories, region-based, phase correlation-based, and feature-based stitching. In our application, we selected feature-based stitching because it is comparatively less time-consuming and much better for ordering an unordered sequence of images [3]. We implement the feature-based automatic stitching with an additional preprocessing algorithm (the precapture feature detection which is part of Sense-Panorama) for high-resolution and robust performance with limited mobile resources. The first step in image stitching is feature extraction and matching, followed by random sample consensus (RANSAC) for inlier and outlier detection. For each pair of matching images, we obtain a set of features that are geometrically consistent called RANSAC inliers, as well as a set of features called outliers that are not geometrically consistent but remain within the overlapping area [4].

#### 2.1.1. Feature Extraction

In feature-based stitching, feature selection is challenging and significant. For the feature points in both images to be matched and similar, the feature points must have enough information and must provide different perspectives, viewpoints, and illumination conditions [5,6].

SIFT

Scale-invariant feature transform (SIFT), which is the most well-known feature detection–description algorithm was introduced by D. G. Lowe in 2004 [4]. Feature points are detected by searching local maxima using difference-of-Gaussians (DoG) at various scales of the subject images. For the SIFT detector, the DoG operator is computed, which is equivalent to the Laplacian-of-Gaussian (LoG). The method extracts a 16 × 16 neighborhood around each detected feature and further segments the region into sub-blocks, manifesting a vector of 128 bin values. SIFT is computationally expensive but strongly invariant to image scaling, rotations, and limited affine variations [4,7].

ORB

The ORB algorithm is a combination of the normalized Binary Robust Independent Elementary Feature (BRIEF) and modified Feature from Accelerated Segment Test (FAST) description methods [7]. FAST corners are computed and detected in each layer of the scale pyramid. Harris corner score evaluates the cornerness of the detected points to filter out the top points. The drawback of the BRIEF description method is that such descriptions are highly unstable with rotation. To overcome this drawback,

AKAZE

The authors in [8] introduced the Accelerated-KAZE (AKAZE) algorithm, which is also based on nonlinear distribution filters such as KAZE, but its nonlinear scale-spaces are built using an efficient computer framework called Fast Explicit Diffusion (FED). The AKAZE detector is based on the Hessian Matrix. Scharr filters are used to improve the matrix rotation invariance quality, and the maxima of the detector responses in spatial areas are considered feature points. The AKAZE definition is based on the Modified Local Difference Binary (MLDB) algorithm, which also works very well. AKAZE features do not change on the scale, rotation, and limited affine transforms and have additional variations at various scales due to nonlinear scale-spaces [7].

#### 2.1.2. Feature Matching

The feature matching scheme adopted in the study is based on the Nearest Neighbor Distance Ratio (NNDR) used by D.G. Lowe to match SIFT features in “Distinctive image features from scale-invariant keypoints” [4] and by K. Mikolajczyk in “A performance evaluation of local descriptors” [9].

In this feature matching procedure, the nearest neighbor and second nearest neighbor of each element (from the first element set) are searched in the second element set. Eventually, the ratio of the nearest neighbor to the second nearest neighbor is calculated for every feature descriptor, and a certain threshold is set to find good matches. The value for the threshold is kept between 0.7 and 0.5 for the best matches. The Sum of Absolute Deviation (L1-norm) is used for matching descriptors of SIFT, while the Hamming distance is used for matching descriptors of AKAZE and ORB. For feature matching, several different algorithms can be used, such as Nearest Neighbor, Nearest Neighbor Distance Ratio, and threshold matching [9]. Incorrect matches (or outliers) are inevitable in the feature-matching phase. Therefore, the outlier rejection phase is mandatory for the accurate fitting of the transformation model. Some robust probabilistic models such as Random Sample Consensus (RANSAC) [10], Progressive Sample Consensus (PROSAC) [11], and M-estimator Sample Consensus (MSAC) [8] can be used for outlier rejection in matched features and for fitting the transformation model.

**Figure 1:**
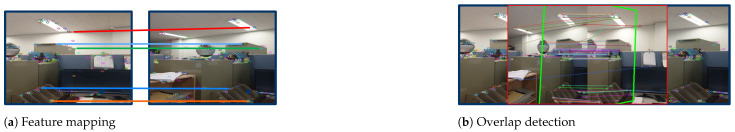
Feature matching. Parallel lines indicate good matches while nonparallel lines indicate bad matches; (**a**) feature matching between two consecutive frames; (**b**) overlap detection between two consecutive frames.

Figure 1 shows how features are matched in the two consecutive frames. Good matches in both frames are represented by parallel lines, and bad matches are represented by nonparallel lines.

#### 2.1.3. Homography Estimation

**Figure 2:**
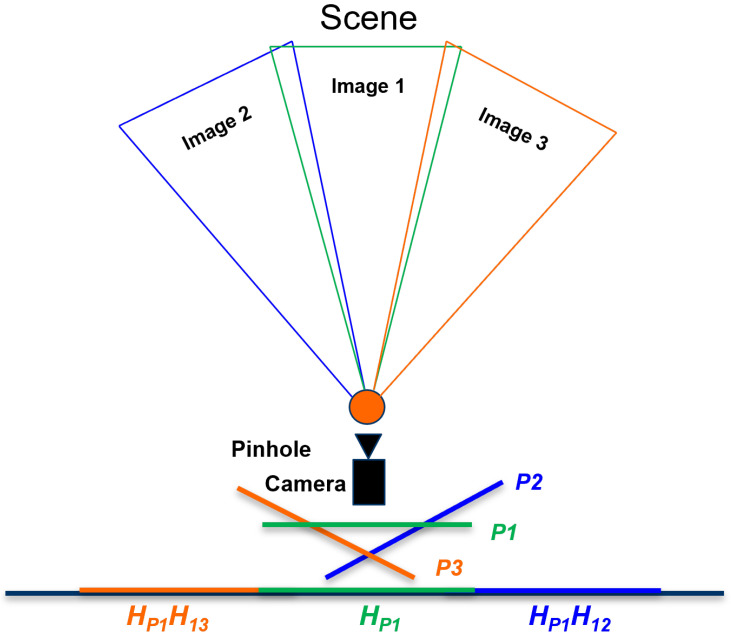
A diagram showing how images that are located on different planes can be related via homography to a common plane, assuming that all the images have a common projection point (a pinhole camera model).

The homography (denoted by H) in computer vision can be defined as a transformation matrix that moves from one plane to another plane through a point of projection. Any two images of the same planer surface in space can be related by homography based on a pinhole camera model, as shown in Figure 2.

In image stitching, homography estimation is required for creating a panorama. Referring to Figure 2, suppose that we want to take pictures of a given scene in a three-dimensional world via a pinhole camera, where all the projections pass through a common point. Let us suppose that we have captured three image frames via a pinhole camera and that all three images are represented in Figure 2 by their names only as Image 1, Image 2, and Image 3. We can see that the first image (Image 1) lies on P1 (plane 1), the second image (Image 2) lies on P2 (plane 2), and likewise for Image 3. All corresponding images that lie on different planes can be related via a homography matrix ($H_{matrix}$) if they share the same center of projection. For the case of a final panorama, all the images must lie on a common plane *P* [4,10] (as shown in Figure 2).

Hence, we can map our first image that lies on P1 to *P* using homography and write it symbolically as $H_{P1}$. Then, we can take a second image that lies on P2, which can be related through *H* and from P2 to P1 as $H_{12}$. Then, multiplying that result with $H_{P1}$ will transform our second image into $P(H_{P1}H_{12})$. Similarly, the third image that lies on P3 (plan 3) can be transformed into *P* by multiplying $H_{P1}$ with $H_{13}$, which will give $H_{P1}H_{13}$. All these transformation relations account for matrix equations that can be solved for nine unknowns from $h_{11}$ to $h_{33}$ (see Equation (1)). The result is a 3×3 matrix representing a set of equations that satisfy the transformation function from the first image plane to the second image plane, as discussed above. For a detailed discussion on homography matrix, see previous studies [12,13,14].

(1) $$H_{matrix}=\left[\begin{array}{ccc} h_{11} & h_{12} & h_{13}\\ h_{21} & h_{22} & h_{23}\\ h_{31} & h_{32} & h_{33} \end{array}\right]$$

#### 2.1.4. Warping and Blending

After successful registration of all images related to each other, warping all the images to a final coordinate space is required. The final coordinate space can be chosen as planar, cylindrical, or spherical depending on the requirements. When all the registered input images are warped in the final coordinate space, the next step is to remove visible seams using blending algorithms [15,16].

#### 2.1.5. Cropping

Usually, the final panorama has irregular boundaries caused by warping, projections, and unwanted camera movements. Automatic cropping is thus applied to remove unwanted irregular boundaries from the final stitched image.

## 3. Proposed Method “Sense-Panorama”

**Figure 3:**
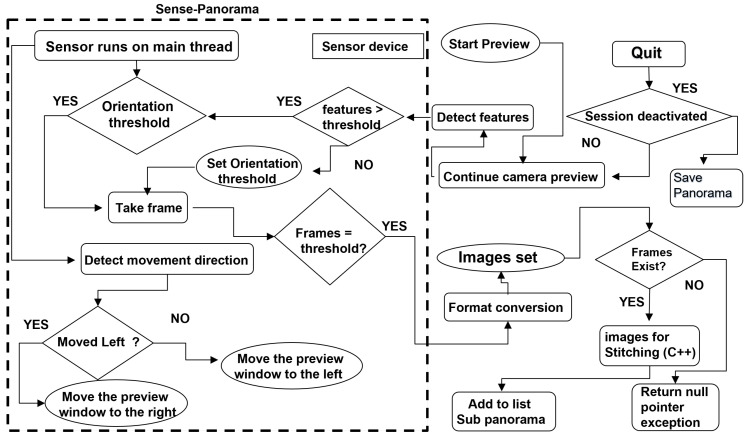
The overall workflow of the Sense-Panorama application.

**Figure 4:**
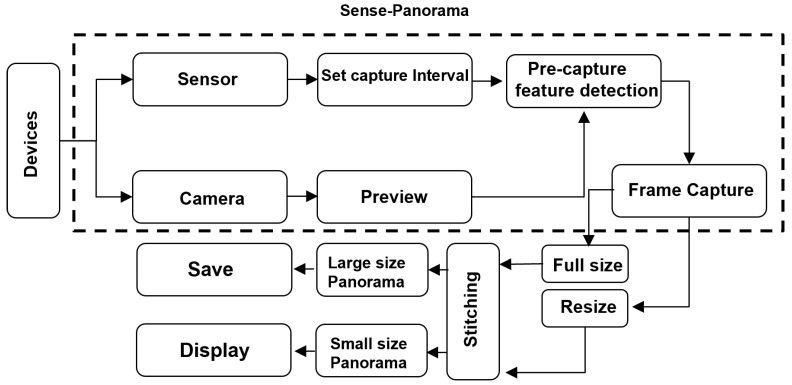
The control flow of the Sense-Panorama method.

In this paper, we presented all the possible solutions to address the issues discussed in the background and proposed a method called, “Sense-Panorama” that results in high-resolution panorama construction on mobile devices. The Sense-Panorama method consists of two main modules, the operation of the orientation sensor and the precapture feature detection (see the highlighted area in Figure 3 and Figure 4). For automatic frame capturing, we have used the orientation sensor that has been discussed in the following subsection. To address stitching failure, we present a precapture feature detection technique in the coming section.

The overall workflow of the panorama application is shown in Figure 3. The highlighted area in this Figure 3 reflects the workflow of the Sense-Panorama method. The orientation sensor is used to detect the movement of the mobile device and automate the frame-capturing process. The direction of the movement is detected as well. The data obtained from the sensor is processed using Equations (2)–(4), to set the threshold for the camera device for frame capturing.

### 3.1. Orientation Sensor

**Figure 5:**
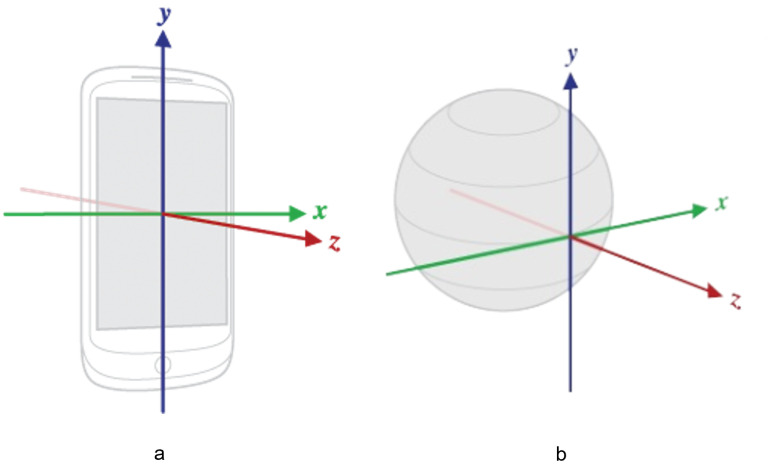
(**a**) Coordinate system (relative to a device) used by the Sensor API.; (**b**) Earth’s coordinate system, a 3-axis system.

Smartphones are equipped with a variety of sensors, one of which is a gyroscope sensor (an orientation sensor). To control costs, Mobile devices are usually equipped with low-quality inertial measurement unit (IMU) sensors. Thus, the gyroscope must be calibrated because gyroscopic errors can magnify exponentially during the integration of the corresponding signals with the computed rotation [17]. This calibration has already been done by Android developers (refer to the documentation for using the orientation sensor in Android devices [18]). Generally, either a 3×3 rotation matrix or a quaternion can be used to represent the rotation of a mobile device. Here, we use a vector matrix called a motion vector [19]. Equations (2)–(4) provide the values for angular acceleration at a given time. The values returned by the smartphone device from the orientation sensor indicate the instantaneous angular acceleration. The Earth’s coordinate system is also a 3-axis system (relative to the surface of the Earth), as seen in Figure 5b [20]. The *y*-axis points to the magnetic north along the surface of the Earth, the *x*-axis is perpendicular to *y* and points east, the *z*-axis points toward space, and the negative *z*-axis point inwards toward the ground.

A reference for the rotation values along the three-coordinate axis can be obtained from the developer’s documentation for a particular smartphone device. The values for all three axes can be used to calculate the amount of device rotation. Thus, using the following equations, we can sense the orientation of the device:

(2) $$X=X+f\times y_{rad}$$

(3) $$Y=Y+f\times x_{rad}$$

(4) $$V_{m}=\left[\begin{array}{c} x_{rad}\\ y_{rad}\\ z_{rad} \end{array}\right]$$

**Figure 6:**
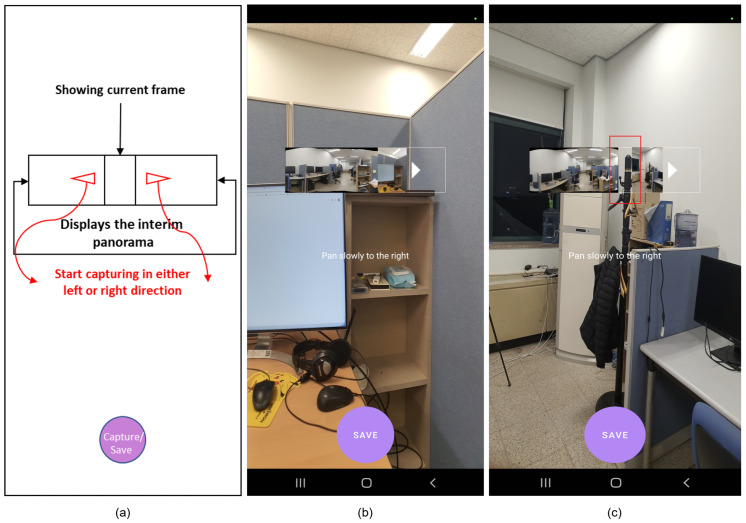
(**a**) GUI’s sketch of the panorama application; (**b**) GUI of the panorama application with the Sense-Panorama method, indicating successful stitching is in progress; (**c**) GUI of the panorama application without the Sense-Panorama method, the highlighted red box indicates discontinuation due to stitching failure.

Equations (2) and (3) are used to sense the device rotation along the *Y*-axis and *X*-axis, respectively, and Equation (4) provides the device’s instantaneous acceleration values along the three axes used in Equations (2) and (3). The variable *Y* returns a floating value, which is then compared to the preset threshold value by the program to capture the next image frame. The value of *f* is constant and equal to 0.3, this value is used to cover the screen width with respect to the user’s 360-degree rotation. In the case of a screen width of 1080, the value of *f* is equal to 360/1080. Each degree movement of the user around the *Y*-axis covers 3 pixels on the screen’s window that shows the current frame (Figure 6a). $X_{rad}$ is the acceleration along the *X*-axis, $Y_{rad}$ is the acceleration along the *Y*-axis and $Z_{rad}$ is the acceleration along the *Z*-axis. These three values are returned by the motion vector $V_{m}$. Similarly, the value of *X* is calculated from Equation (2). This value is used to detect and control the unwanted motion of the device along the *Y*-axis (parallel to the *y*-axis), which may cause misalignment or stitching failure.

### 3.2. Precapture Feature Detection

During the preview, a frame from the camera device is taken, and the number of features in this current preview frame is detected and compared with the predefined threshold value (see Table 1 for the minimum number of features that must be present in a frame), such that a successful overlap area can be detected with the next frame. If too few features are present in the current frame, the next frame is captured before reaching the predefined frame capture interval using the device’s orientation sensor; the interval for the frame capture is set by the orientation sensor. This approach ensures successful overlap area detection and, eventually, successful stitching. Pseudocode for the “Sense-Panorama” is presented in Section 3.3. Figure 4 shows the workflow for the orientation sensor with the precapture feature detection technique. The sensor is used to detect the device orientation and set the interval for the image frame capture, while the camera device provides the frame for the feature detection module. The feature detection module detects the number of features and sets the interval for the orientation sensor. The sensor then captures the image frame when the interval is reached.

### 3.3. Pseudo-Code for Sense-Panorama

Algorithm 1 shows the pseudocode of the Sense-Panorama method that can be used to capture frames at the right time to ensure that no stitching failure occurs.
**Algorithm 1** Sense-Panorama Algorithm.**Require:** Frames from image analysis**Ensure:** Frame is captured with the aided sensor **while** panorama capturing session is on **do**      **if** Sensor value == threshold value **then**          capture frame          **if** number of captured frames are greater than 2 **then**                generate the interim panorama                display the interim panorama          **end if**      **else if** Sensor value != threshold value **then**           get frame from image preview analysis           detect features           set threshold for the next frame capture      **end if****end while**

Another method can be used to avoid stitching failure, where a fixed and predefined sufficient offset area (overlap) is set between the two images. However, this approach will lead to an excess amount of overlapping area in each frame, which will increase the computational costs.

### 3.4. Misalignment Correction and Triggering Events

**Figure 7:**
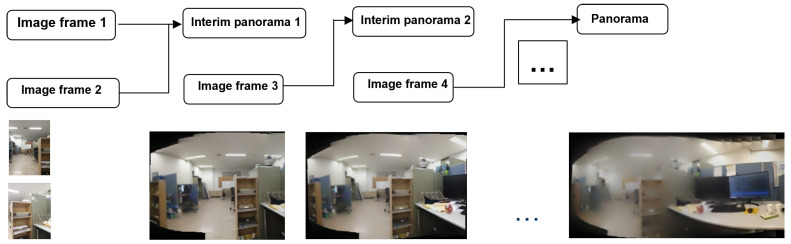
Distortion in the panorama when sequential stitching is performed.

The orientation sensor is used to detect the device movement for all three axes, as shown in Figure 5b. During the panorama capture session, any movement in the vertical direction (parallel to the *y*-axis; see Figure 5a) during the capture is undesirable, as movement can cause the misalignment of frames during the capture session. Misalignment of consecutive frames causes unwanted darker regions in the final panorama and, in the worst cases, can cause stitching failure. Another issue is distortion in the panorama. Distortion occurs because when two images are stitched together, during stitching in the warping phase, the images are transformed from their destination plane into a new target plane due to their homography relationship, as discussed in Section 2.1.3. In the sequential stitching method, during the automatic capture session, two frames are initially stitched to form an interim panorama. This interim panorama is then stitched with the next captured frame to form a new interim panorama and so on. Stitching after every frame capture results in distortion. Figure 7 visualizes the flow of sequential stitching. We implemented the following method to address this issue:

- If the overlap area increases when using the precapture feature detection technique, do not stitch unless four image frames are being captured.
- Otherwise, perform stitching only if two image frames are captured at the specified normal interval by the sensor.
- Retain the current interim panorama to be stitched together with the next frame.

**Figure 8:**
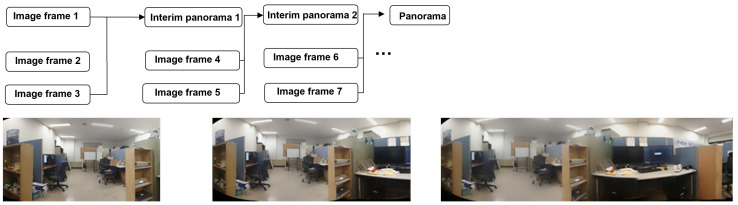
A distortion-less panorama in sequential stitching when Sense-Panorama method is used.

Following this approach, no distortion appears in the sequential stitching method. Figure 8 shows the results of our approach for distortion removal. A low-resolution panorama is generated as a display to the user. At the same time, a high-resolution panorama proceeds in parallel, which is then saved at the end of the panorama capturing process. Four main modules need to be executed simultaneously on the CPU of the smartphone. The issue of deadlock and memory overflow has been addressed by categorizing our program’s modules into asynchronous and synchronous tasks. These modules are listed below (also refer to Table 2 and Section 3.3 for implementation details).

- Module 1: Camera device and sensor device on the user interface.
- Module 2: Precapture feature detection module.
- Module 3: Interim panorama stitching.
- Module 4: Full-sized panorama stitching.

**Table 2:** Implementation details of the different modules.

Asynchronous Tasks (C++)	Synchronous Tasks (Java)
Pre-capture feature detection	Frame Capturing (Camera Device)
Panorama Stitching (for storage)	Orientation detection (Sensor Device)
Interim panorama stitching (for display)	User Interface

The first module (module 1) executes the camera device and sensor device on the user interface thread synchronously with each other; the camera module captures frames; and the sensor module triggers events, controls camera capture events, and moderates the stitching process. The remaining three modules in the above list are executed asynchronously, and their results are returned to the main thread when they are ready.

Figure 6a shows the graphical user interface of our Android-based panorama application. The main rectangle in the middle of the screen is used to display the interim panorama to the user. This rectangular box is shown by double arrows. The small window in the middle of the rectangular box is used for displaying the current image frame to the user. When the user starts capturing, this small window starts moving in the direction of the motion caused by the user’s movement.

Figure 6b shows a screenshot of the panorama application during the capture session. When Sense-Panorama is used, the process successfully proceeds with no stitching failure and the interim panorama is displayed to the user as can be seen in the rectangular box of Figure 6b.

Figure 6c shows another screenshot of the panorama application. When the application is used without the Sense-Panorama, stitching failure occurs, and the interim panorama is not updated. The rectangular box shows that there is a discontinuation of the stitching process as highlighted by the small vertical rectangle in red. The comparison of Figure 6b with Figure 6c shows that with the aid of the Sense-Panorama, the panorama capturing shot proceeds without failure. Another issue that has been successfully addressed by our Sense-Panorama is that of deadlock and memory overflow. When the submodules were executed according to the procedure listed in Table 2, the application responded without any deadlock and memory overflow. Without the Sense-Panorama the application could not proceed and was abandoned by the Android OS.

## 4. Experimental Results

**Figure 9:**
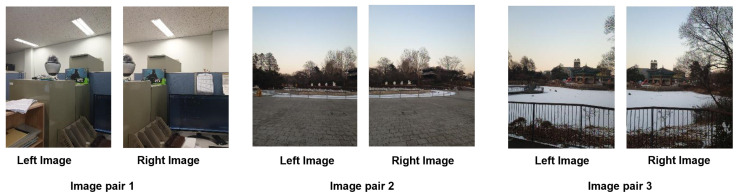
Shows the 3 pairs of images, with each pair containing two images (left image and right image with a sufficient overlap area).

**Figure 10:**
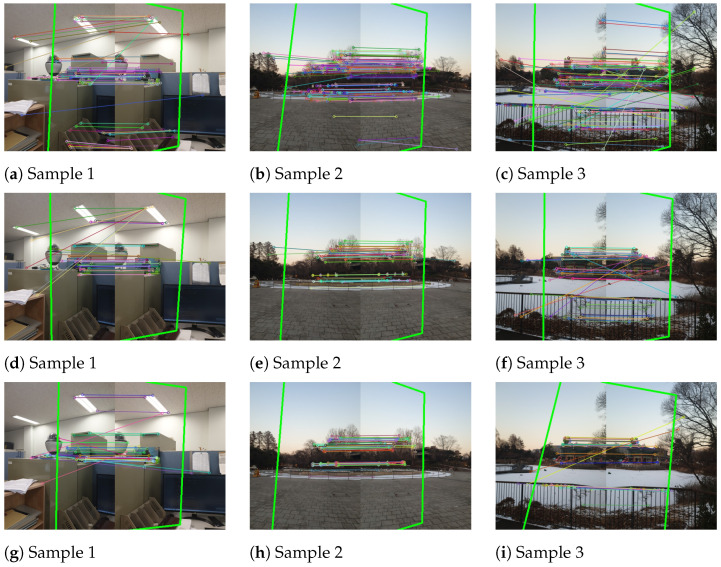
Three different image pairs were tested for overlap area detection with SIFT, AKAZE, and ORB: (**a**–**c**) show the overlap area detected by the SIFT algorithm in image pair 1, image pair 2, and image pair 3, respectively; (**d**–**f**) show the overlap area detected by the AKAZE algorithm in image pair 1, image pair 2, and image pair 3, respectively; and (**g**–**i**) show the overlap area detected by the ORB algorithm in image pair 1, image pair 2, and image pair 3, respectively.

**Figure 11:**
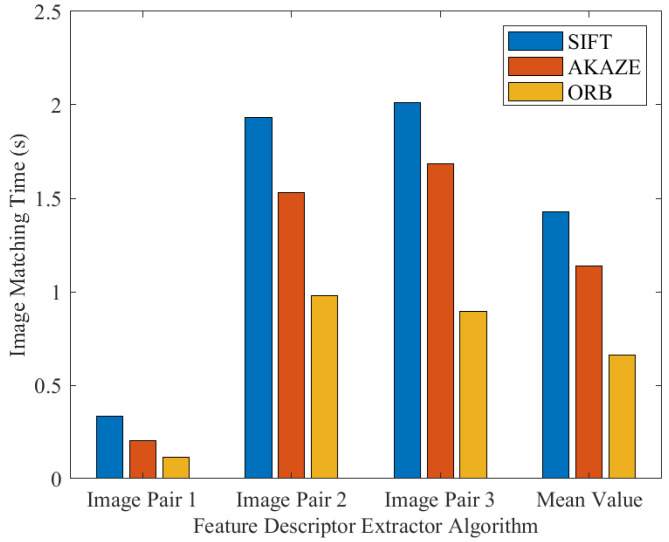
A comparison of the computational costs of SIFT, AKAZE, and ORB.

To compare the performance and computational costs of the above-mentioned feature detection algorithms (SIFT, AKAZE, and ORB), we used a test dataset containing a total of 20 pairs of images (each image in the image pair has been resized to a resolution of 604 × 403 during the program execution) (The dataset can be downloaded from https://www.kaggle.com/datasets/yaseenksk/dataset-panorama). The data generated from this dataset are presented in Table 3. The image matching test was implemented in C++, and we recorded the following information: (a) the number of feature detected by each algorithm in each pair of images (each pair of images contained a left image and a right image); (b) the total number of feature matched in each pair of images; (c) the total number of inliers detected in each pair of images; (d) and the time taken by each algorithm to match each pair of images. Data from only three image pairs are shown in Table 3. These three image pairs can be seen in Figure 9, and their resulting matched images are also shown in Figure 10 (which shows how these image pairs were matched using the SIFT, AKAZE, and ORB algorithms). Additionally, Figure 11 visualizes the data shown in Table 3.

Figure 9 shows the three image pairs selected from the above-mentioned dataset.

All three feature detector algorithms (SIFT, AKAZE, and ORB) were tested for feature detection and image matching. The highlighted green lines (Figure 10a–i represent the overlap area between the two consecutive image frames detected by the three aforementioned algorithms. The SIFT algorithm was used for image matching as shown in Figure 10a–c; the AKAZE algorithm was used for image matching as shown in Figure 10d–f; and, similarly, the ORB algorithm was used for image matching as shown in Figure 10g–i. Figure 10g–i show that the fewest feature were detected by the ORB algorithm.

Figure 11 compares the computational costs of the SIFT, AKAZE, and ORB algorithms in the image-matching process (i.e., the time taken by each algorithm to detect and match features in each pair of images). The results clearly show that the SIFT algorithm is computationally more costly, while AKAZE is slightly faster than SIFT, and ORB is the fastest. The results generated in all the experiments that we have presented here in this paper are executed by Samsung Galaxy SM-N960N.

From the dataset of 20 image pairs, we also computed the total number of matches and misses (in the matching process for each pair of images) performed by each algorithm, as shown in Table 4. The criterion for matching is to successfully detect an overlap area between a given pair of images, Figure 10 shows successful overlap area detection by SIFT, AKAZE, and ORB algorithm. Table 4, shows that ORB is the poorest performer in feature detection and image matching, while SIFT is the most accurate among the ORB and AKAZE algorithms, having correct matches of up to 94%. AKAZE is approximately 1/3 times less accurate than SIFT. Based on our study (Figure 11), for feature extraction and image matching [9,21], we tested the SIFT, ORB, and AKAZE algorithms and recommend using SIFT (Other feature detectors can also be used like BRIEF, LBP, and HOG key points. It’s totally a developer’s choice. Our main goal is to present the Sense-Panorama method to solve the issue of stitching failure and panorama distortion which occurs in sequential panorama application development.) as the most suitable feature detector for image matching if accuracy is desired [2,7]. In cases where speed is of the essence, then ORB can be used while compromising accuracy. Hence, to avoid stitching failures on mobile devices, SIFT and AKAZE can be used with more confidence than ORB [7].

The available datasets for panorama generation have frames captured at larger time differences. However, our method utilizes a real-time capture mechanism. Hence the available dataset was not suitable for evaluation. Therefore, we performed live capture of 30 test dataset frames including scenes from indoors, outdoors textured, and texture-less (The dataset can be downloaded from https://www.kaggle.com/datasets/yaseenksk/dataset-panorama (accessed on 10 December 2022)). We captured the frames at 4032 × 3024 resolution. The application was installed on a Samsung Galaxy SM-N960N, which carries SoC as Qualcomm Snapdragon 845 and a CPU of 4 × 2.8 GHz Kyro 385, the RAM size is 8 GB and is 1866 MHz. The Sense-Panorama is compared with Brown [22], Bouguet [23], and panorama applications on a current smartphone camera. The method in [23] is implemented on a high resolution (4032 × 3024) frame and is based on RANSAC outlier removal.

### 4.1. Performance Comparison

**Figure 12:**
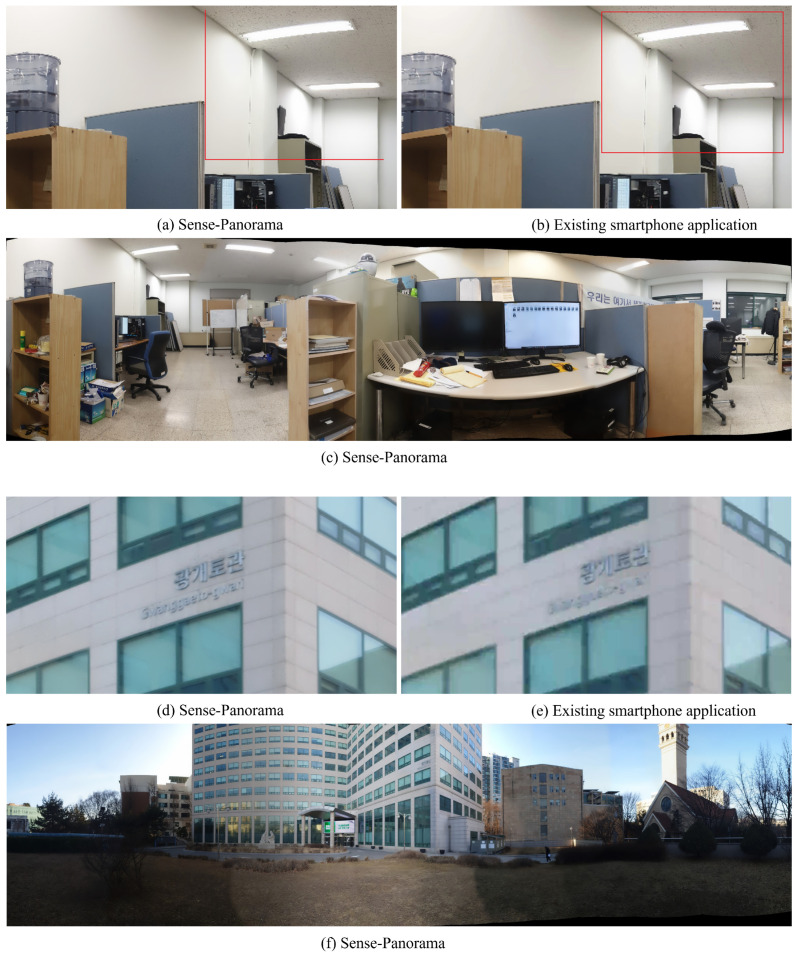
(**a**,**b**) show a comparison between Sense-Panorama and the existing smartphone application, (**c**) is a complete panoramic image shot taken by Sense-Panorama. Similarly (**d**) and (**e**) show a comparison between Sense-Panorama and the existing smartphone application, (**f**) is a complete panoramic image shot taken by Sense-Panorama.

Figure 12c shows a high-resolution panorama generated by Sense-Panorama. Figure 12a,b shows the comparison of Sense-Panorama with that of the existing smartphone application. Figure 12a indicates a 4-times zoomed-in cropped region from the panorama generated by Sense-Panorama. Figure 12b indicates a 4-times zoomed-in cropped region from the panorama generated by the existing smartphone application. The comparison between, Figure 12a,b clearly indicates that (a) retains its high quality compared to (b). This comparison also holds true if we look at the results in Table 5 and Table 6.

A similar comparison has been done between Figure 12d,e. Figure 12f is the panorama generated by Sense-Panorama. It can be seen clearly that, Sense-Panorama has the ability to generate a high-resolution panorama with high quality.

### 4.2. Subjective Evaluation

Visual quality evaluation was performed by 6 persons, to find out the registration and stitching artifacts. An image is termed as “good” if no major artifacts are observed using a 32” monitor at 100% zoom. A quality score was calculated using Equation (5):

(5) $$\mathrm{Quality}\mathrm{Score}=\frac{\mathrm{number}\mathrm{of}\mathrm{good}\mathrm{images}}{\mathrm{total}\mathrm{number}\mathrm{of}\mathrm{images}}$$

Table 5 shows the quality score results of the Sense-Panorama method with the existing smartphone application. The results clearly indicate that the Sense-Panorama outperforms the smartphone panorama application.

### 4.3. Objective Evaluation

Quantitative analysis was performed by two matrices, Structural Similarity Index (SSIM) [24] and Peak Signal to Noise Ratio (PSNR). The stitching error is calculated in the overlapped area between the input image and the generated panorama. Table 6 presents the results and depicts that the Sense-Panorama is able to generate a high-resolution panorama with good quality.

In this study, an automatic–sequential, high-resolution panorama stitching method (Sense-Panorama) for mobile devices was developed, implemented, and presented. Sense-Panorama was tested indoors, outdoors, and under different lighting conditions. Sense-Panorama performed very well both outdoors and indoors. However, the performance was much better when creating panoramas outdoors. The precapture feature detection approach ensured successful feature-based high-quality stitching with limited resources on mobile devices.

**Figure 13:**
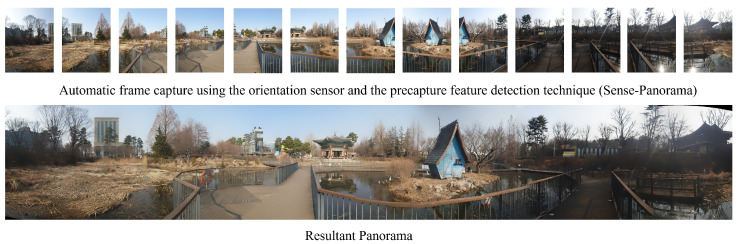
Shows the individual frames captured by Sense-Panorama and its corresponding generated panoramic image.

The present approach uses an automatic sequential procedure for panorama capturing. Each source image acquired during the capturing session is stitched to the interim panoramic image with the aid of a precapture feature detection module and an orientation sensor that sets the interval for the frame capture. This procedure ensures that the next frame has sufficient features (see Table 1) for successful stitching. Sense-Panorama also ensures a high-resolution panorama with the minimum number of frames being captured (Figure 13), thus reducing the burden of extra computational costs.

Sense-Panorama has been implemented using C++ and the mobile device’s native programming language. All the image processing tasks, such as feature detection, feature matching, homography estimation, and stitching, were implemented using C++ (OpenCV), and the remaining tasks, such as running the sensor device, camera device, and user interface, has been implemented using native languages. In our case, we used Java for Android devices. Furthermore, these tasks must be categorized and implemented as asynchronous and synchronous tasks during implementation. Table 2 shows the list of synchronous and asynchronous tasks in our program. The synchronous tasks need to be executed in the foreground process while asynchronous tasks are executed in the background as a separate thread, enabling smooth and successful execution of the algorithm.

## 5. Conclusions

Sense-Panorama method for automatic panorama generation is a sequential stitching method. Which involves automatic frame capture using the orientation sensor and the precapture feature detection method. The frames are sequentially stitched and the interim panorama is displayed on the screen, the method is very efficient as this process only requires the current interim panoramic image and the current source image(the latest frame captured by the camera device) to generate the next interim panorama, while the rest of the images are discarded. This feature makes it possible to run the program on mobile devices with little memory consumption. Future work will include further reducing the average stitching time interval and reducing memory consumption by reusing homography matrix values obtained from smaller-sized images only once in the stitching process for corresponding high-resolution image frames. The SIFT algorithm is the most suitable feature descriptor detector (Other feature detectors can also be used like BRIEF, LBP, and HOG key points. It’s totally a developer’s choice. Our main goal is to present the Sense-Panorama method to solve the issue of stitching failure and panorama distortion which occurs in sequential panorama application development.) for sequential stitching-based panorama image generation. Combining SIFT and AKAZE features for image stitching may enable a further increase in accuracy [25].

## Figures and Tables

**Table 1:** The minimum number of features required (threshold values) for successful image matching.

Image Resolution	SIFT	AKAZE	ORB
Low	200	200	200
High	500	500	480

**Table 3:** Quantitative comparison and performance costs of the given feature-detector descriptor.

Algorithms	Features Detected in the Image Pairs		Features Matched	Inliers	Matching Time(s)
	Left Image	Right Image			
Dataset Image Pair 1					
SIFT	2665	3498	719	373	0.335
AKAZE	2418	2884	382	267	0.203
ORB	489	501	191	81	0.113
Dataset Image Pair 2					
SIFT	9308	5688	1012	506	1.934
AKAZE	2325	6422	689	379	1.532
ORB	506	516	327	227	0.981
Dataset Image Pair 3					
SIFT	6352	5094	913	539	2.013
AKAZE	4465	4090	623	346	1.683
ORB	515	506	281	131	0.896
Mean Values for all Image Pairs					
SIFT	6108.3	4760	881.3	472.6	1.427
AKAZE	3069.3	4465.3	564.6	330.6	1.139
ORB	503.3	507.6	266	146.3	0.663

**Table 4:** The number of correct matches among the 20 pairs of images when using SIFT, AKAZE, and ORB.

	SIFT	AKAZE	ORB
Matches	18	11	7
Misses	2	9	13
Matches [%]	90	55	35

**Table 5:** Subjective quality evaluation on captured data using a quality score.

Dataset	Size	Sense-Panorama	Existing Mobile Panorama App
Textured images	20	0.95	0.87
Texture-less images	10	0.91	0.74

**Table 6:** Objective quality matrices comparison.

Method	SSIM	PSNR
Brown [22]	0.923	32.3
Bouguet [23]	0.963	44.2
Existing smartphone application	0.972	46.3
Sense-Panorama	0.985	47.8

## Data Availability

Not applicable.
